# The soil microbiomes of forest ecosystems in Kenya: their diversity and environmental drivers

**DOI:** 10.1038/s41598-023-33993-4

**Published:** 2023-05-02

**Authors:** Lorine Akinyi Onyango, Florence Atieno Ngonga, Edward Nderitu Karanja, Josiah Ochieng’ Kuja, Hamadi Iddi Boga, Don A. Cowan, Kennedy Wanjau Mwangi, Marianne Wughanga Maghenda, Pedro Bixirao Neto Marinho Lebre, Anne Kelly Kambura

**Affiliations:** 1 Department of Biochemistry, Jomo Kenyatta University of Agriculture and Technology, P. O. Box 62000-00200, Nairobi, Kenya; 2 International Centre for Insect Physiology and Ecology, P.O. Box 30772-00100, Nairobi, Kenya; 3 Department of Botany, Jomo Kenyatta University of Agriculture and Technology, P. O. Box 62000-00200, Nairobi, Kenya; 4 School of Agriculture, Earth and Environmental Sciences, Taita Taveta University, P. O. Box 635-80300, Voi, Kenya; 5 Centre for Microbial Ecology and Genomics, Department of Biochemistry, Genetics and Microbiology, University of Pretoria, Pretoria, South Africa; 6 International Livestock Research Institute, P.O. Box 30709-00100, Nairobi, Kenya

**Keywords:** Computational biology and bioinformatics, Ecology, Microbiology, Molecular biology

## Abstract

Soil microbiomes in forest ecosystems act as both nutrient sources and sinks through a range of processes including organic matter decomposition, nutrient cycling, and humic compound incorporation into the soil. Most forest soil microbial diversity studies have been performed in the northern hemisphere, and very little has been done in forests within African continent. This study examined the composition, diversity and distribution of prokaryotes in Kenyan forests top soils using amplicon sequencing of V4-V5 hypervariable region of the 16S rRNA gene. Additionally, soil physicochemical characteristics were measured to identify abiotic drivers of prokaryotic distribution. Different forest soils were found to have statistically distinct microbiome compositions, with *Proteobacteria* and *Crenarchaeota* taxa being the most differentially abundant across regions within bacterial and archaeal phyla, respectively. Key bacterial community drivers included pH, Ca, K, Fe, and total N while archaeal diversity was shaped by Na, pH, Ca, total P and total N. To contextualize the prokaryote diversity of Kenyan forest soils on a global scale, the sample set was compared to amplicon data obtained from forest biomes across the globe; displaying them to harbor distinct microbiomes with an over-representation of uncultured taxa such as *TK-10* and *Ellin6067* genera.

## Introduction

Forests are highly productive components of terrestrial ecosystems^1^, covering more than 40 million km^2^ and presenting 30% of the total global land area^1^. They form part of our most precious natural resources essential to the continued balance and survival of the world’s ecosystem^2^. Forests act as carbon sinks where soil organic matter is formed from residuals after biomass decomposition^1^. They play a major role in the global cycling of carbon, as well as organic nitrogen mineralization and conversion of organic phosphorus into inorganic^3^. Moreover, forests are involved in maintenance of soil structure^1^, organic matter decomposition^3^, degradation of pollutants^4^ and shape soil microbial communities through the symbiotic interaction with primary microbial producers such as mycorrhizal fungi^5^. Some of bacterial taxa previously shown to dominate forest soil ecosystems and play such key roles include members of the genera *Pedobacter* and *Chitinophaga* (*Bacteroidetes*); *Pseudomonas*, *Variovorax*, *Ewingella*, and *Stenotrophomonas* (*Proteobacteria*)^1^; *Burkholderia*, *Phenylobacterium*, and *Methylovirgula* (*Pseudomonadota*)^6^; members of the *Rhizobiales* and *Nitrosopumilus*^7^. Unfortunately, these forest ecosystems have suffered from serious depletion due to anthropogenic activities associated with over-farming, the pulp and paper industry and population encroachment into peri-urban areas, along with other land-use change^6^. Soil microorganisms are an important component of the forest ecosystem^1,5^ as they play fundamental roles in most nutrient transformations within forest soils, upon which the stability and sustainable development of forest ecosystems rely^8^.

The distribution and diversity of soil microbiomes is influenced by numerous aspects such as soil type, physicochemical characteristics, microclimate, vegetation and land-use^6^. Recent microbial ecology studies have shown that different habitats harbor diverse microbial communities whose succession patterns are shaped by substrate availability, including nutrient pools, physiochemistry and vegetation^5^.

In addition, factors that modify the microclimate and forest litter chemistry such as forest type, plant species and plant diversity have also been identified as drivers of microbial community composition in forest soils^7^.

Kenya’s indigenous forests represent some of the most diverse ecosystems in the world, and provide important economic, environmental, recreational, scientific, cultural and spiritual benefits to the nation (Republic of Kenya, 2009)^9^. Forests play a vital role in the stabilization of soils and ground water, support the conduct of reliable agricultural activity and play a crucial role in protecting water catchments in Kenya besides moderating climate by absorbing greenhouse gases^10^. In addition, forests such as those of the Taita Hills are hotspots of biodiversity, harboring a wide variety of medicinal plants^11^. The Forests Act has previously recognized that forests provide the main locus of Kenya’s biological diversity and a major habitat for wildlife, and acknowledges that forests and trees are the main source of domestic fuelwood^2^. However, these forests have been subjected to land-use changes such as conversion to farmlands, ranches and settlements.

Historically, the majority of forest soil microbial diversity studies have been performed in northern hemisphere countries, with very little focus on the forests of the African continent, even in global studies^9,10^. To fill this knowledge gap, this study aimed to document the microbial ecology of selected Kenyan forest soil ecosystems, and to study the possible abiotic drivers. The selected forest ecosystems are among Kenyan landscapes endowed with varied climate with different water catchment and soil regime. For instance, the Mt. Kenya, Aberdare and Taita Taveta regions are among Kenya’s water towers. The regions are characterized by a bimodal rainfall patterns which influence the vegetation within each ecoregion. This leads to a variation in moisture content within soil ecosystems further influencing microbial diversity.

## Results and discussion

### Different forest soils in Kenya have unique physicochemical properties

**Figure 1:**
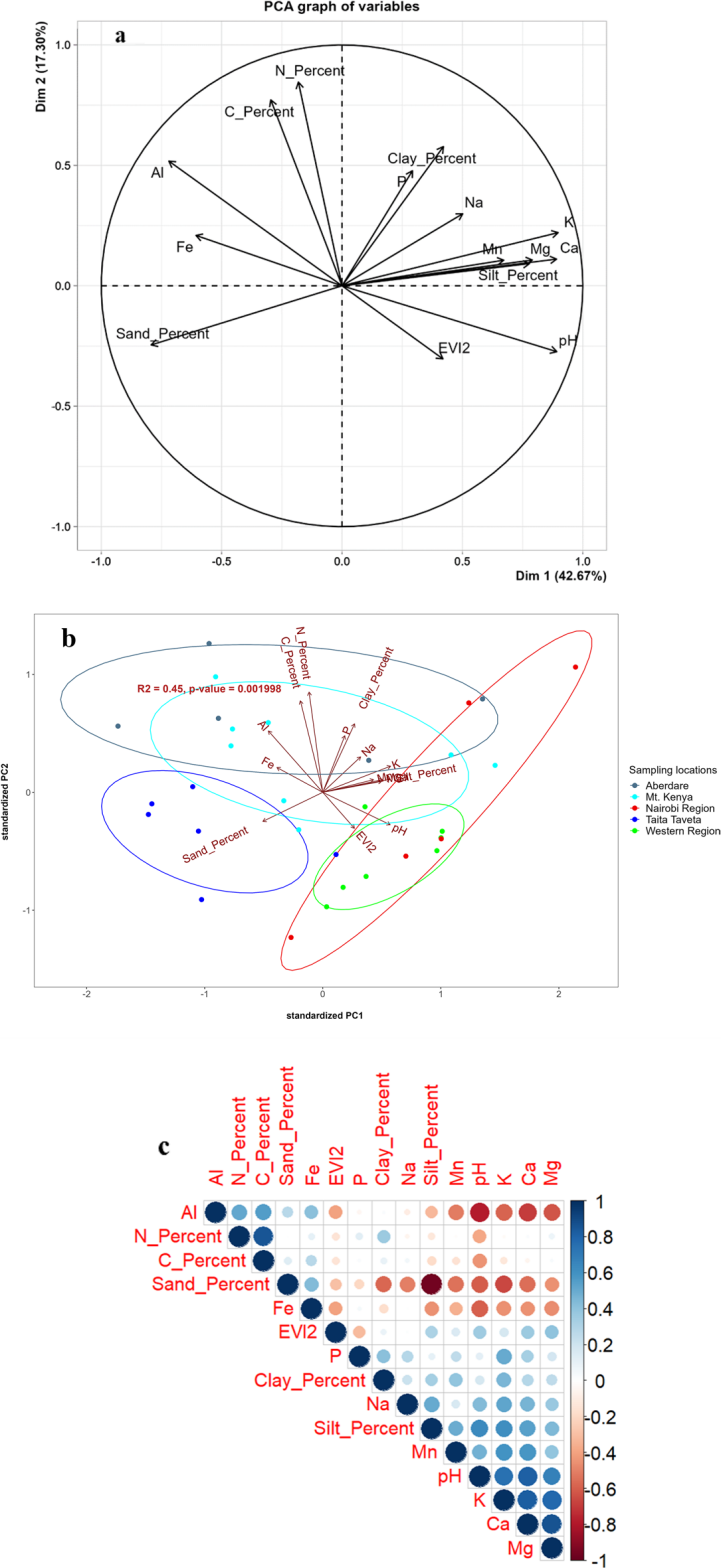
(**a**) Principal component analysis (PCA) biplot of forest soil samples according to their physicochemical properties. The influence of each variable on sample distribution is represented by the arrows radiating from the center of the PCA plot. (**b**) The sample clusters corresponding to different ecoregions are highlighted within ellipses of the same color. (**c**) Pearson correlation plot between measured soil physicochemical properties. Positive and negative correlations are displayed in blue and red shades, respectively, while the size and intensity of matrix circles is proportional to correlation coefficient between variables.

In this study, 31 soil samples were obtained from forests ecosystems within the Taita Taveta, Nairobi, Western, Aberdare and Mt. Kenya ecoregions (Supplementary Table 1). Samples from the different ecoregions were shown to be significantly different (p-value ≤ 0.01, R^2^ = 0.45) in terms of soil physicochemical properties, specifically in soil pH, soil texture, macro- and micro-nutrient composition and Enhanced Vegetation Index-2 (EVI2) (Fig. 1a and b, Supplementary Fig. S2a and S2b). Taita Taveta forest soils were highly distinct from those of the Nairobi, Aberdare and Western regions (Fig. 1b). Conversely Nairobi and Western region soils exhibited the least variability (Fig. 1b).

Several soil physicochemical properties were found to be correlated, and thus could be considered as interdependent (Fig. 1c). For instance, the measurement of plant density, vegetation index (EVI2), was positively correlated with all the measured soil nutrients, apart from phosphorus. This is not unexpected, as nutrient-rich forest soils have been repeatedly shown to support high density plant growth^12^. The soil samples used in this study were collected from 0 to 5 cm depth, which is within the 0–20 cm soil profile characteristically comprising the organic horizon that results from decomposition of litter-derived organic matter and representing a nutrient-rich mixture of processed, plant-derived organic matter^12^. Low titratable phosphorus concentrations were possibly due to presence of a high content of Al and Fe, which form oxides that fix phosphorus at the low pH’s associated with these soils^13^. In this study, the pH was positively correlated with EVI2 but negatively correlated with C and N content. This result contradicts a previous study that concluded that at higher soil pH levels, the mineralizable fractions of C and N increased, possibly due to the direct effect of pH on microbial populations and their activities^13^.

### Taxonomic composition of soil microbiomes across Kenyan forest biomes

**Figure 2:**
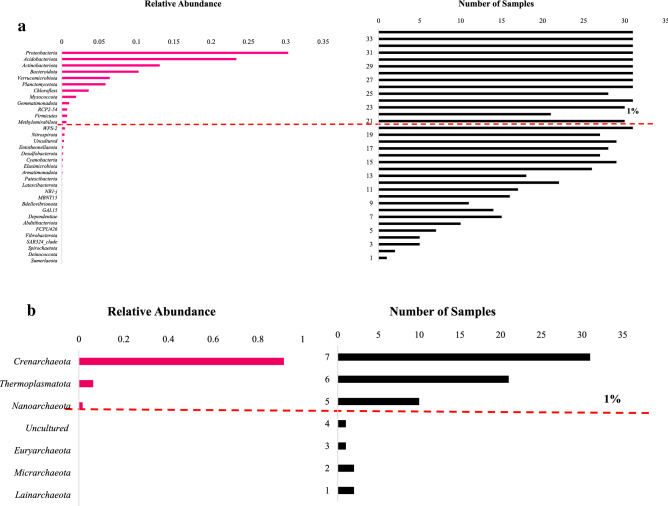
((**a**) and (**b**)) Mean relative abundances of prokaryotic ((**a**) bacteria) ((**b**) Archaea) phyla across forest soil samples, together with the number of samples in which they were identified. The phyla scoring > 1% mean relative abundance, are dominant and above red dashed line.

Analysis of Bacterial diversity in forest soil samples indicated the presence of 34 phyla, of which 12 were dominant (i.e. represented by > 1% of ASV reads in at least 87% of the samples). The most abundant of these was *Proteobacteria* (30.3% mean relative abundance), followed by *Acidobacteriota* (23.4% mean relative abundance) and *Actinobacteria* (13.1% mean relative abundance) (Fig. 2a). *Actinobacteriota* members such as *Frankiales* and *Streptomycetales* are known as nitrogen-fixing bacteria and may produce biologically active secondary metabolites^14^*.* The dominant bacterial phyla from the current study were consistent with other studies within two forests sites, where bacterial ASVs were assigned to 44 phyla, ten of which; (*Proteobacteria*, *Acidobacteria*, *Verrucomicrobia*, *Firmicutes*, *Actinobacteria*, *Bacteroidetes*, *Planctomycetes*, *Chloroflexi*, WD272, and *Gemmatimonadetes*) comprised more than 90% of the relative abundance in each library^15^. Our results on bacterial abundance were also consistent with several previous studies where *Proteobacteria*, *Acidobacteria*, *Verrucomicrobia*, *Firmicutes*, *Actinobacteria*, *Bacteroidetes*, *Planctomycetes*, *Chloroflexi* were the most abundant phyla^15^.

In particular, members of *Proteobacteria* and *Acidobacteriota* phylum have been reported as ubiquitous and dominant in` soil ecosystems^16^. Members of these Phyla, such as *Anaeromyxobacter, Bradyrhizobium*, *Azospirillum, Ralstonia, Burkholderia, Brevundimonas Rhodopseudomonas* (Proteobacteria), *Mycobacterium, Nocardia*, A*mycolatopsis Thermobispora*, *Pseudonocardia*, *Brachybacterium*, *Frankia*, *Conexibacter* (Actinobacteria), *Streptococcus*, *Lactococcus*, and *Enterococcus* (Firmicutes) have been reported to carry out various key ecological processes such as regulation of biogeochemical cycles, decomposition of biopolymers, exopolysaccharide secretion and plant growth promotion^17^.

The most dominant taxa at Order level, *Rhizobiales* (12.8% mean relative abundance), *Burkholderiales* (6.3% mean relative abundance) and *Chitinophagales* (6.2% mean relative abundance), were represented across all samples (Supplementary Table 2). The order *Chitinophagales* contains members that are known to degrade complex organic matter, such as chitin and cellulose^18^. The orders *Rhizobiales, Xanthomonadales and Rhodospirillales* found in this study are also well known for nitrogen fixation, mineralization and denitrification activities^7^. *Crenarchaeota* was the most abundant Archaeal phylum represented across all samples, with 91.6% mean relative abundance (Fig. 2b). This phylum was further grouped into two classes; *Nitrososphaeria* (77.1% mean relative abundance) and *Bathyarchaeia* (0.2% mean relative abundance). *Nitrososphaeria* includes many ammonia-oxidizing taxa that have been identified previously in forest soil microbiomes^19^. Other phyla within the Archaeal Domain included *Thermoplasmatota* (6.4% mean relative abundance), represented within about two thirds of the soil samples, (Supplementary Table 2) while *Nanoarchaeota* phylum (1.7% mean relative abundance) was represented within about a quarter of the soil samples. These results agree with previous studies where archaeal communities in forest biomes were reported to be dominated by *Nitrososphaera*^20^. Members of *Nitrososphaera* have been described as major contributors to soil biogeochemical processes such as carbon, methane, nitrogen and, sulfur cycle within many ecosystems^21^.

### Alpha- and beta- diversity analysis of soil prokaryotic communities

**Figure 3:**
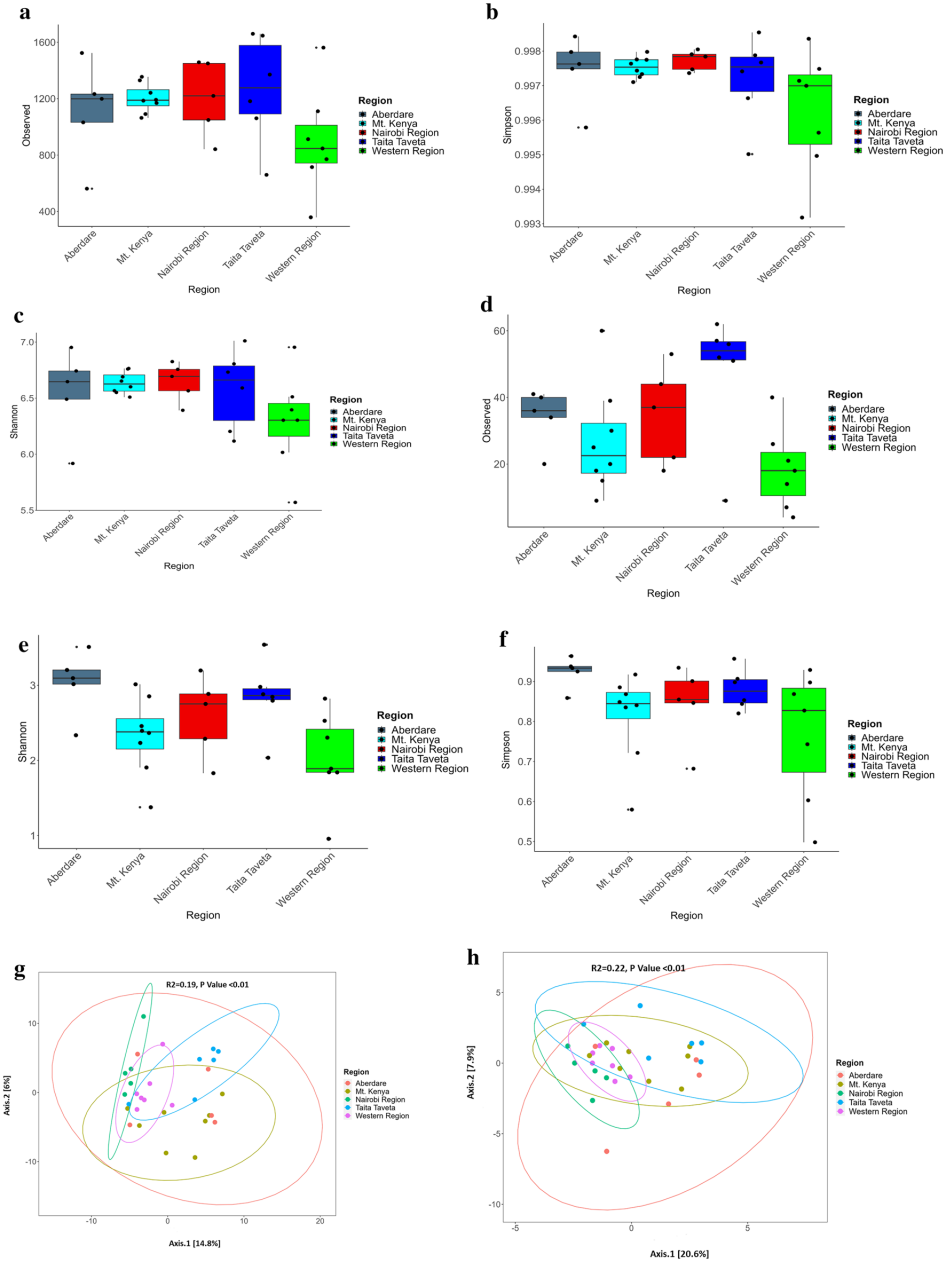
(**a–f**) Alpha Diversity of soil prokaryotic communities. (**a–c**) represent diversity indices (Observed, Shannon and Inverse Simpson) of Bacterial communities while (**d–f**) represent diversity indices (Observed, Shannon and Inverse Simpson) of Archaea communities within soil samples collected from the five regions in Kenya. Figure 3 (**g,h**) Principal Coordinate Analysis of prokaryotic diversity based on Bray–Curtis index within soil samples. (**g**) represents beta diversity of Bacterial community structure while (**h**) represents beta diversity of Archaea community structure within soil samples collected from the five regions in Kenya.

Analysis of sample alpha-diversity showed Western and Taita Taveta regions soils to have significantly different (P ≤ 0.01) levels of Archaeal richness, while Western and Aberdare regions soils displayed significantly different Shannon diversity index (P = 0.02) **(**Fig. 3d–f). Although there were no significant differences between bacterial communities displayed within various forests ecosystems **(**Fig. 3a–c**)** soil samples under bamboo vegetation cover within Mt. Kenya and Aberdare regions displayed lower diversity than the other ecoregions. Soils collected from the Taita Taveta region (Vuria and Ngangao) were shown to have the highest number of observed prokaryotic taxa. These forests are characterized by a montane climate vegetation with thick ground cover^22^. The high number of ASVs could be attributed to a broad range of bacterial micro-habitats associated with high nutrient availability besides other specific microbial diversity drivers such as plant density and vegetation index that positively influenced bacterial abundance^23^. There was high prokaryotic variability observed within each region, an indication of distinct microhabitats and microclimates in each forest region covered (Fig. 3 g,h).

Beta-diversity analysis of soil samples from these regions showed a significant difference (P < 0.01) on bacterial and archaeal community structure (Bacteria *R*^*2*^ = 0.22; Archaea *R*^*2*^ = 0.24) (Fig. 3 g,h). Notably, the microbial composition of samples from the Taita Taveta region showed a lower degree of overlap with other regions, which mimics the soil chemistry differences observed between the regions. Taita Hills comprise the northernmost part of the Precambrian Eastern-Arc Mountain range, known for its rich biodiversity^24^ and recognized as one of the world’s 25 biodiversity hot-spots^25^.

The highly significant (P < 0.01) richness and Shannon diversity index values for samples from Western region forests could be attributed to the tropical nature of forests within this region such as sample K21 (Kakamega forest) which is considered an important biodiversity reservoir and the only remaining Guinea-Congolian tropical rain forest in Kenya^26^. Kakamega forest is the largest moist lowland forest ecosystem in Kenya, and has similar characteristics to Central Africa equatorial forests^22^.

**Figure 4:**
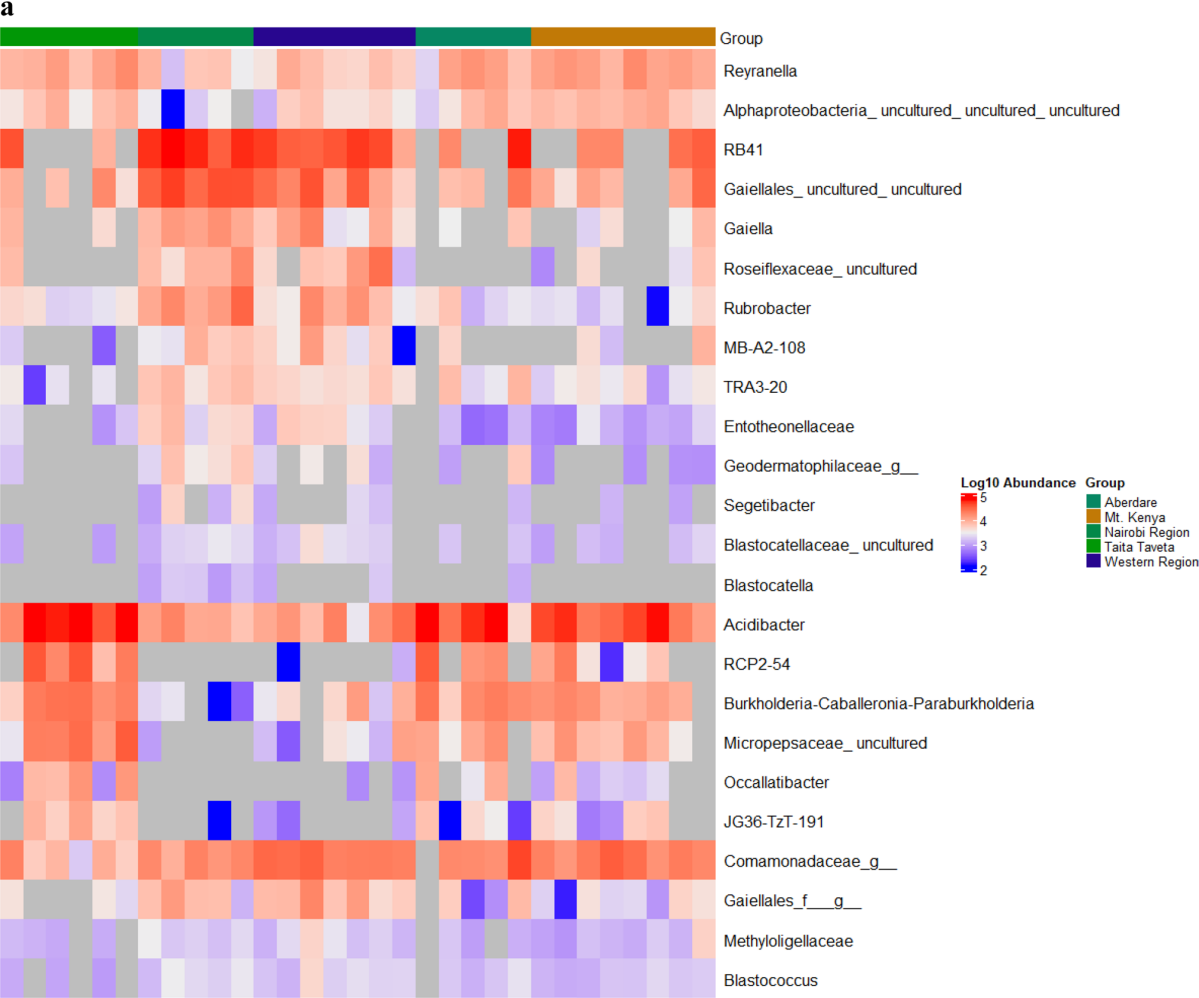

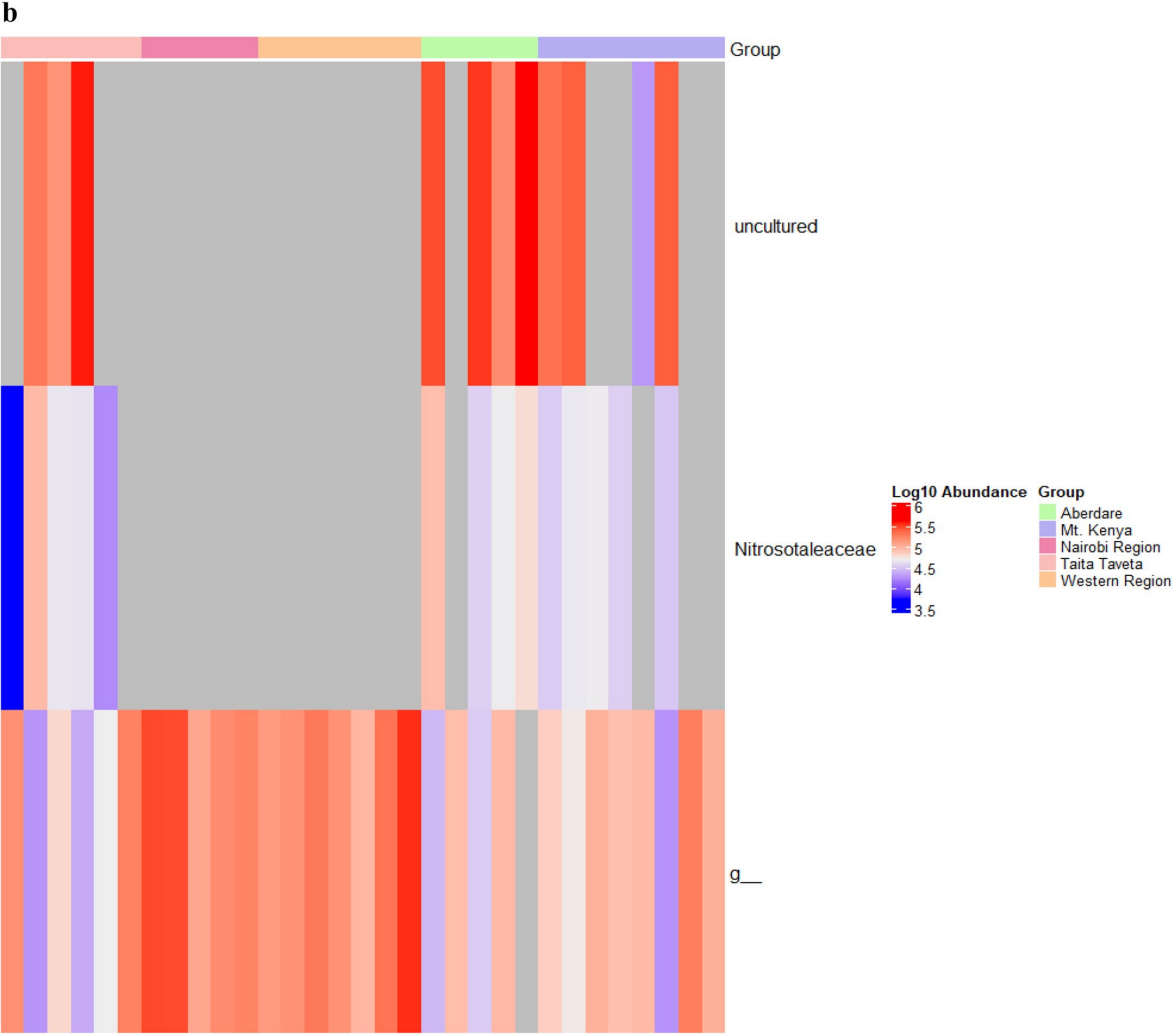
Taxa, at genus level, that are over-represented in different regions based on the LEfSe analysis. (**a**) represents LEfSe analysis of Bacterial communities while (**b**) represents LEfSe analysis of Archaea communities within soil samples collected from the five regions in Kenya. The log_10_ abundance colour scheme represents LDA score. The colors represent the group in which that taxon was found to be more abundant compared to the other groups. f_ indicates that the ASV was not able to be classified to a family while g_ indicates that the ASV was not able to be classified to a genus. The heat maps were generated using Microbiome Marker R package^56^.

To explore further the differences in the soil microbiome structure between the different forest areas, linear discriminant analysis (LDA) effect size (LEfSe) was used to detect prokaryotic taxa that were differentially abundant within and between soil samples. In a comparison of samples from the five forest regions (Aberdare, Mt. Kenya, Nairobi, Taita Taveta and Western), several taxa were identified as differentially abundant (P adj. < 0.01): 13 genera in Taita Taveta, 21 in Nairobi, 1 in Mt. Kenya, 2 in Western and 5 in Aberdare region (Fig. 4a). The LEfSe algorithm identified several differentially abundant archaeal taxa (P adj. < 0.01) within the three regions (Aberdare, Nairobi and Taita Taveta) each having a taxon (Fig. 4b). The genus *Acidibacter* was over-represented in Taita forest soils, possibly due to the low soil pH observed in this region. *IMCC26256* was over-represented in western region. *Burkholderia-Caballeronia-Paraburkholderia taxa,* which typically have a very broad ecological diversity and metabolic versatility^27^ were the most abundant in Aberdare Forest soils*, RB41* in Mt. Kenya while *Rhodovastum* was the most abundant in Mt. Kenya region soil samples (Fig. 4a).

### Environmental drivers of soil microbiomes in Kenyan forest soils

**Figure 5:**
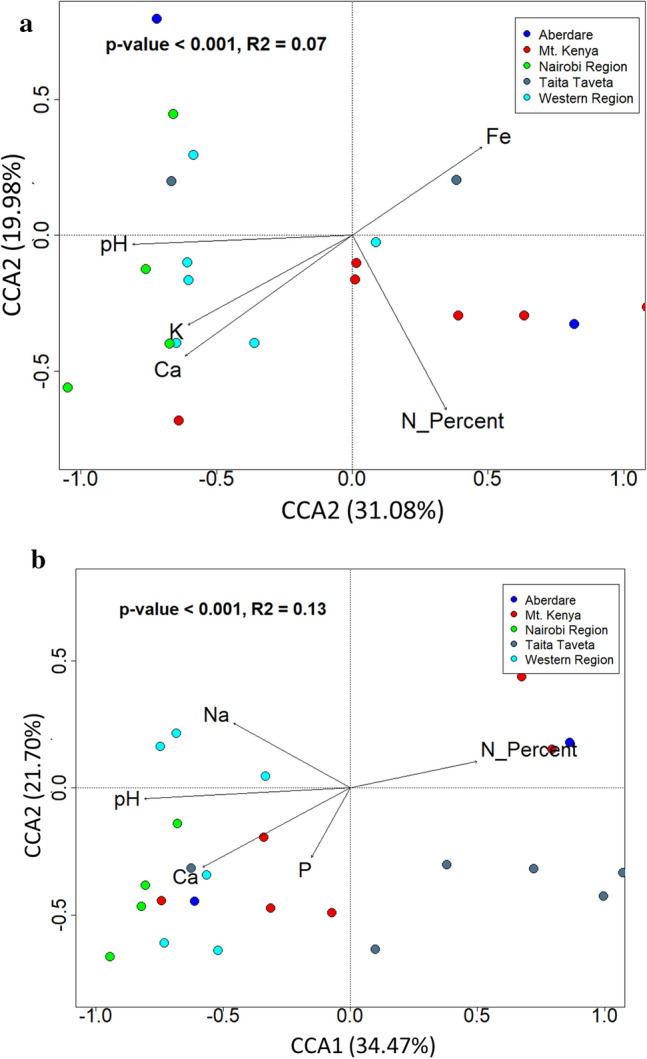
(**a,b**) Canonical correspondence analysis (CCA) plots showing the effect of soil physicochemical characteristics and plant density index on bacterial and archaeal communities at 99% significance. The percentage explained by various soil characteristics is expressed in the CCA1 and CCA2 axes and samples were color-coded on the plots according to forest regions. (**a**) represents CCA of Bacterial communities while (**b**) represents CCA of Archaea communities within soil samples collected from the five regions in Kenya.

A stepwise model-building approach for constrained ordination models was used to assess the potential environmental drivers of the prokaryotic communities within forest ecosystems. Canonical correspondence analysis (CCA) ordination plots showed that bacterial and archaeal community structures were significantly affected by several soil physicochemical characteristics (P < 0.01). Soil pH, Ca, K, Fe and %N were shown as key drivers of bacterial community structure, while Na, pH, Ca, P and %N were important factors in shaping archaeal community structure within forest soils (Fig. 5a,b). The significant effect of nitrogen to community structure is consistent with the composition of soil microbiomes described in this study, which were dominated by taxa potentially involved in nitrogen fixation such as *Cyanobacteria* and *Nitrospirota* (Supplementary Fig. S2a). Fe concentration and soil texture are known to be major factors in shaping bacterial community structures in some soils^28^. Soil pH possibly affected the thermodynamics and kinetics of microbial respiration, thus shaping the microbial communities’ composition and function.

### The “uniqueness” of Kenyan forest microbiomes

**Figure 6:**
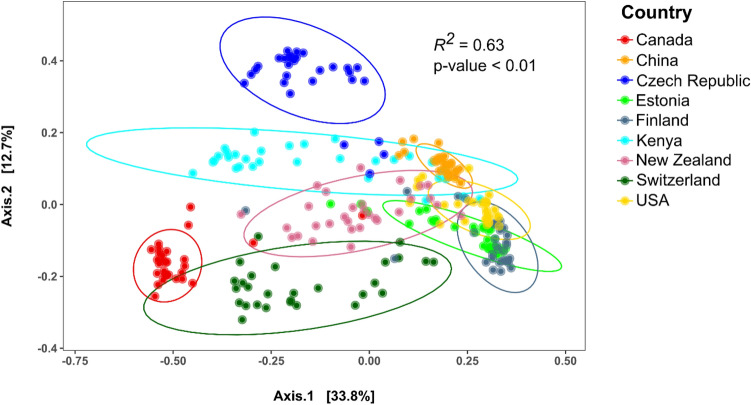
Principal Component Analysis (PCoA) ordination of the Bray–Curtis distance between samples from different country datasets. Samples are colored according to country of origin. The PERMANOVA significance results on differences in beta-diversity according to country of origin are displayed in the plot. Ellipses around the clusters of samples represent the predicted distribution of points within each country group at a 95% confidence interval.

In order to address the question of whether Kenyan forest soils harbor unique microbiome compositions, the phylogenetic datasets used in this study were compared with datasets on forest soil microbiomes from other countries across the globe **(**Supplementary Table 3**)**. Comparisons of the beta-diversity scores between these datasets, based on Bray–Curtis index (Fig. 6), revealed community structures of forest soil microbiomes which were, to some extent, distinguishable by the country of origin (*R*^2^ = 0.63; p-value < 0.01). The Kenyan dataset formed a distinct group with some degree of overlap with soil microbiomes from China, the Czech Republic, New Zealand and the USA. This overlap could be a result of common plant cover between the sampled areas in the different countries. Some forests in Kenya are known to harbor globally distributed plant species such as bamboo (*A. alpina*), indigenous plant species found within forests with highest floral diversity such as (*Coffea fadenii*, *Juniperus procera*—African pencil cedar, *Podocarpus falcatus*, *latifolius*, *Tabernaemontana stapfiana*, *Ocotea usambarensis*, *Macaranga conglomerata*, and *Psychotria petit*. Forests harboring moderate floral diversity included *Podocarpus*, *Dombeya*, *Croton megalocarpus*, while dryland species included Acacia species such as *A. tortilis*, *A. melifera*, *A. abyssinica, and A. polyacantha*. Plantation species included *Eucalyptus grandis*, *E. saligna*, *E. camaldulensis* and *E. urophylla*^29^. It is also worth noting that the Kenyan dataset exhibited the highest variability of beta-diversity scores, which reflect the variety of ecoregions sampled in this study.

The significant compositional differences between national datasets were reflected in the LDA comparison results, which identified 178 taxa differently distributed across the datasets (Supplementary Table 4). Fourteen of these were over-represented in Kenyan forest soils, including the Archeal genus *Nitrososphaera*. Other over-represented genera of potential ecological relevance to Kenya forest soils included *Bradyrhizobium*, which is positively associated with soil health^30^ and *Chitinophaga*, members of chitinolytic Myxobacteria known to control fungal populations in soils^31^. It is also worth noting that several of the over-represented taxa in the Kenyan soil dataset belonged to uncultured groups of bacteria, including members of uncultured genera *TK-10* and *Ellin606,* an indication of Kenyan forest soils may harbor a catalogue of novel taxa. During development of bio-conservation strategies in these forest regions, consideration of these distinct microbiomes with unique taxa should be taken into account.

## Materials and methods

### Study site and sample collection

This study was part of an ongoing consortium project that focused on a primary-scale survey of soil chemistry and microbiology across a range of regional and climatic zones in sub-Saharan Africa^9^.

**Figure 7:**
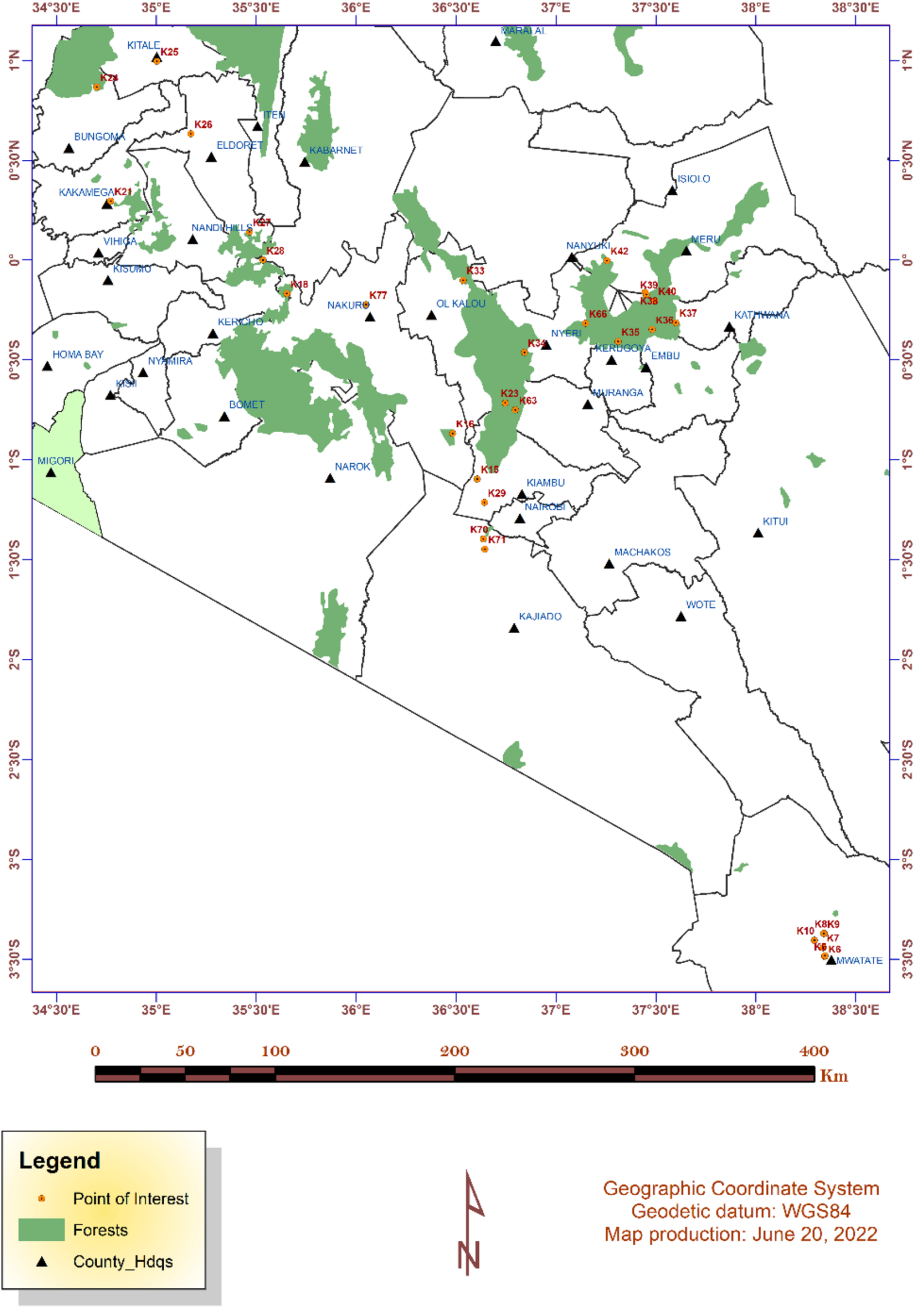
Kenyan forest sites where soil samples were collected. The samples are indicated sequentially from K5 to K77 within the map. The point of interest represents each sampling site. The map was created using ArcGIS 10.8.1 (Environmental Systems Research Institute software application, 2020); https://www.esri.com/en-us/arcgis/products/arcgis-platform/overview.

In Kenya, a microbiome survey of the soils across selected forest ecosystems was carried out based on a census for forest regions (http://kws.go.ke/content/overview-0). Data capture at each sampling site included GPS location, elevation, vegetation at the time of sample collection, slope, general soil description and general site description. To accurately show the sampled forest sites to scale, a map was constructed using the GPS coordinates captured from the forests during fieldwork using ArcGIS 10.8.1 (Environmental Systems Research Institute software application, 2020), https://www.esri.com/en-us/arcgis/products/arcgis-platform/overview; which was used to visualize and display the sample sites. The layers for towns, rivers, lakes and roads were added from ArcGIS Online database to enrich the thematic map as shown in (Fig. 7). The distribution and characteristics of the selected forests used in this study are summarized in Supplementary Table 1.

Sampling was done by recovering 4 × 200 g topsoil samples (0–5 cm depth) at approximately 50 m spacing at each site. Each working sample was obtained by scooping a composite of 4 × 50 g pseudo-replicate samples, recovered from the corners of a one square meter virtual quadrat. Each sample was collected in a separate labelled Whirl Pak bags and stored at 4 °C prior to shipment to University of Pretoria (South Africa) for nucleic acid extraction and soil physicochemical analysis.

These samples were later grouped into regions depending on geographical location on the Kenyan map as follows: Aberdare (Sample K23, K33, K34, K63 and K77); Mt. Kenya (K35, K36, K37, K38, K39, K40, K42 and K66); Nairobi (K15, K16, K29, K70 and K71); Taita Taveta (K5, K6, K7, K8, K9 and K10) and Western region (K18, K21, K24, K25, K26, K27 and K28).

### Soil physicochemical characteristics

Soil physicochemical characteristics (Supplementary Table 1) were determined using protocols outlined by AgriLASA (2004). Soil pH was measured using the slurry method at a 1:2.5 soil/water ratio, and the pH of the supernatant was recorded with a calibrated bench top pH meter (Crison Basic, + 20, Crison, Barcelona, Spain). The concentrations of soluble and exchangeable of sodium (Na), potassium (K), carbon (C), magnesium (Mg), and phosphorus (P) were determined using Mehlich 3 test^32^. The extractable ion concentration was quantified using ICP-OES (Inductively Coupled Plasma Optical Emission Spectrometry, Spectro Genesis, SPECTRO Analytical Instruments GmbH & Co. KG, Germany). Soil particle size distribution (sand/silt/clay percent) was measured using the Bouyoucos method^33^. Total nitrogen (TN) and soil organic carbon (TOC) were measured using the catalyzed high temperature combustion method (Dumas method)^34^. The Enhanced Vegetation Index-2 (EVI2) was obtained from the NASA Land Processes Distributed Active Archive Center’s (LP DAAC) VIIRS Vegetation Indices dataset^35^ at a 500-m resolution.

### Prokaryotic DNA extraction and 16SrRNA gene sequencing

Total DNA was extracted from soil samples using the DNeasy PowerSoil Kit (QIAGEN, USA) following the manufacturer's instructions with the following modifications; the elution buffer C6 was pre-heated to 55ºC for 10 min before the final elution step, and the DNA was eluted using 70 μl of the elution buffer. After extraction, DNA concentration and purity were checked using the Nanodrop 2000 (ThermoFisher, USA) and agarose gel electrophoresis. The DNA samples were sent to MRDNA laboratories (www.mrdnalab.com, Shallowater, TX, USA) for sequencing of the V4/V5 16S rRNA gene, using the 515F (5'-GTGYCAGCMGCCGCGGTAA-3') and 909 R (5'-CCCCGYCAATTCMTTTRAGT-3') primers, according to^9,36^. Before library preparation, the regions of interest were amplified using the HotStarTaq Plus Master Mix Kit (Qiagen, USA) and subsequently purified using calibrated Ampure XP beads (Beckman Coulter Life Sciences, USA). Sequencing was performed at MR DNA (www.mrdnalab.com, Shallowater, TX, USA) on MiSeq instrument following the manufacturer’s guidelines.

### Sequence analysis and taxonomic classification

The generated raw amplicon sequence reads were filtered, trimmed, and clustered into unique amplicon sequence variants (ASVs) using the QIIME2 pipeline^37^. Briefly, raw sequences were demultiplexed, quality checked and a feature table constructed using Divisive Amplicon Denoising Algorithm 2 (DADA2) pipeline^38^ inbuilt within QIIME2^39^.The raw sequences were denoised and chimeras removed. Sequences which were < 200 base pairs after phred20- base quality trimming, with ambiguous base calls, and those with homopolymer runs exceeding 6 bp, were removed. The forward and reverse reads were truncated at 324 base pairs. This was followed by calculation of denoising statistics, picking of representative sequences and creation of ASVs feature table. Sequencing processing resulted in a total of 1,944,316 high quality sequence reads, which were clustered into 41,901 ASVs at 3% genetic distance.

Representative sequences were aligned using MAFFT^39^ and highly variable regions were masked to reduce the noise in phylogenetic analysis^40^. Phylogenetic trees were created and rooted at midpoint on QIIME2. Taxonomic classification of ASVs was done using QIIME feature-classifier^39^ against the untrained SILVA 138.1 (release 2022.2)^41^. Demultiplexed high-quality sequence reads were deposited in the National Centre for Biotechnology Information (NCBI) Sequence Read Archive (SRA), as Bio Project ID: PRJNA851255 and study accession numbers available for download at http://www.ncbi.nlm.nih.gov/bioproject/851255. In addition, the metadata, soil chemistry data, input files Qiime and R analysis scripts were deposited at https://zenodo.org/ and a DOI-10.5281/zenodo.7827433 available using the link; https://doi.org/10.5281/zenodo.7827432.

### Data processing of amplicon datasets from other countries.

Sequence datasets from selected forests around the globe were downloaded from publicly available databases (accession numbers at Supplementary Table 3) and processed using the QIIME2 pipeline as described above. Raw reads from the downloaded datasets spanned the 16S rRNA gene hypervariable regions v3-v4, v4, and v4-v5, depending on the study. To keep the sample sizes between countries comparable, a subset of between 28 to 30 samples was chosen for each dataset. To accommodate the variable quality scores of the different datasets, quality threshold was set to 20 and all reads were truncated at 220 bps. After DADA2 processing, the resulting representative sequence file and ASV table were merged with the Kenyan dataset. Read counts for the combined dataset ranged from 10877 to 346157 reads (Supplementary Fig. S1). The merged representative sequence file was taxonomically annotated using the un-trained SILVA database 138.1 (release 2022.2)^41^.

### Statistical analysis

ASVs from QIIME2 were modified for use with the package phyloseq (version 1.36.0) in RStudio^42^. The taxonomy table was merged with the feature table, and the relative abundance and bar plots were plotted using the ggplot2 package (version 3.3.5)^42^. The normality of the dataset was first tested with the Shapiro–Wilk test^43^. The Kruskal–Wallis Rank Sum test^44^ was subsequently used to calculate the significance of mean differences in soil variables between forest soil samples (adj. p. value < 0.01). Tukey post hoc analysis test^41^ were used to compare significant differences between regions where soil environmental variables were normally distributed (adj. p. value < 0.01).

Significant differences in soil physicochemical characteristics were calculated using the stats package version 3.6.2 in RStudio version 4.0.3^41^. The distribution of soil physicochemical variables across different forest sites was calculated on log-standardized data using the “*decostand*” function from vegan package (version 2.5.7)^45^, which performs principal component analysis of the data (PCA)^45^. The resulting distance matrix between samples was plotted in a PCA graph, with the projected direction and magnitude of the distribution for each variable plotted in a separate loading plot. The hmisc (version 4.5) package^41^ was subsequently used to calculate strong significant Pearson correlations^44^ between variables (adj. p-value < 0.01), which were plotted in a bubble graph using the corrplot (version 0.9) package^41^. Biodiversity metrics (alpha diversity) and community structure dissimilarity (beta diversity) were calculated using the vegan (version 2.5.7)^46^ and phyloseq (version 1.16.2)^47^ packages in RStudio. Observed richness, Inverse Simson^48^ and the Shannon indexes^49^ were used as metrics for alpha-diversity^48^. The prokaryotic ASV table was split into Archaea and Bacteria using the “subset_taxa” function in phyloseq before calculating the diversity indexes. Differences in alpha-diversity between designated regions were assessed as described for the soil physicochemical variables. Beta-diversity index of each soil sample was calculated from the Centered log-ratio transformation (CLR) ASV tables using the “vegdist” function in vegan, based on Bray-Cutis distance estimation method^50^. Ordination of the beta-diversity scores was plotted on a principal component analysis plot (PCoA)^51^, and the significance of beta-diversity dissimilarity between forest regions was calculated using Permutational Multivariate Analyses of Variance (PERMANOVA)^52^ with 999 permutations. Comparison of beta-diversity distribution between the samples of different countries datasets was also performed using the methodology described above.

The environmental drivers of prokaryotic community structure were estimated using Redundancy analysis (RDA)^53^. The soil physicochemical dataset was z-score standardized and tested for multicollinearity using the “vif” function from the car (version 3.0.11) package^54^. The best models for explanatory variables were calculated using forward step-wise regression model selection method with the ordistep() function in the vegan package, with 1000 permutations, and significant variables with vif values above 10 were removed. The significance of the best fitted models and each predictor variables in the model were calculated using the ANOVA permutation test with 1000 permutations^55^. The relative taxonomic abundances of prokaryotic taxa were compared between regions using Linear Discriminant Analysis (LDA) effect size (LEfSe) algorithm^56^ for high-dimensional biomarker discovery and explanation of differentially abundant organisms. This analysis was implemented using the package Microbiome Marker in RStudio^56^. Differences were analyzed using Kruskal–Wallis sum-rank test^57^ to detect significant differentially abundant taxa at genus level (adj. p. value < 0.01). The biological consistency was investigated using a set of pairwise tests among genera using the Wilcoxon rank-sum test^58,59^, with an LDA threshold of 2. The same LDA method was used to detect differently abundant taxa across the country datasets.

## Supplementary Information

Supplementary Figure S1.

Supplementary Figure S2.

Supplementary Figure S3.

Supplementary Table 1.

Supplementary Table 2.

Supplementary Table 3.

Supplementary Table 4.

## Data Availability

The demultiplexed high-quality sequence reads has been deposited in the National Centre for Biotechnology Information (NCBI) Sequence Read Archive (SRA), as Bio Project ID: PRJNA851255 and study accession numbers available for download at http://www.ncbi.nlm.nih.gov/bioproject/851255. This Whole Genome Shotgun project has been deposited at DDBJ/EMBL/GenBank under the Bioproject PRJNA291812. The metadata, soil chemistry data, input files for Qiime and R analysis scripts were deposited at https://zenodo.org/ and a DOI-10.5281/zenodo.7827433 available using the link; 10.5281/zenodo.7827432.
