# Supplementary material for: The soil microbiomes of forest ecosystems in Kenya: their diversity and environmental drivers
Source: Sci Rep. 2023 May 2;13:7156. doi: 10.1038/s41598-023-33993-4 (PMC10154314; doi:10.1038/s41598-023-33993-4)
Supplement: Supplementary file 1 — Supplementary Figure S1. [file 41598_2023_33993_MOESM1_ESM.pptx]

## Slide 1
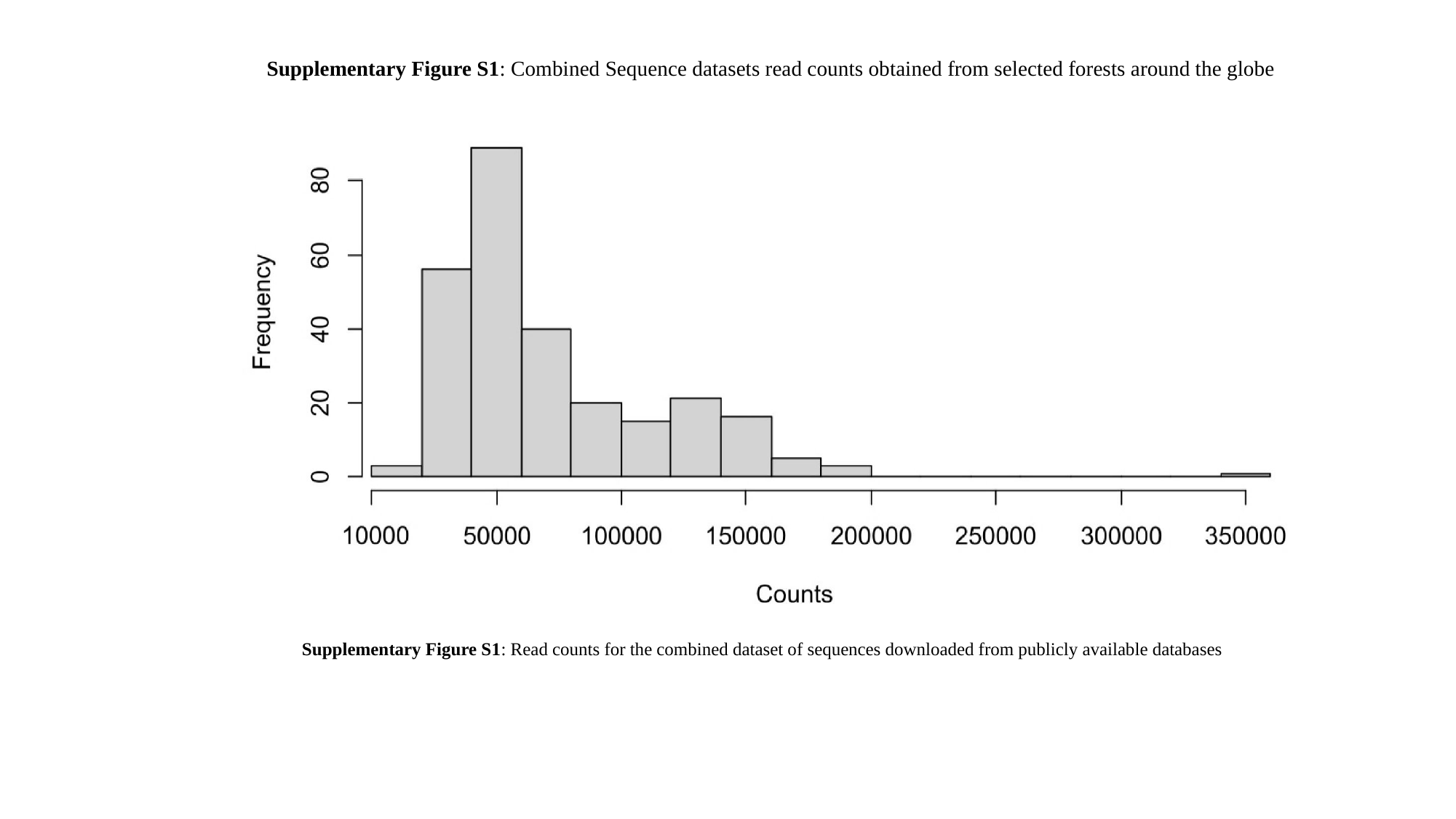

Supplementary Figure S1: Combined Sequence datasets read counts obtained from selected forests around the globe
Supplementary Figure S1: Read counts for the combined dataset of sequences downloaded from publicly available databases
