# Supplementary material for: The soil microbiomes of forest ecosystems in Kenya: their diversity and environmental drivers
Source: Sci Rep. 2023 May 2;13:7156. doi: 10.1038/s41598-023-33993-4 (PMC10154314; doi:10.1038/s41598-023-33993-4)
Supplement: Supplementary file 3 — Supplementary Figure S3. [file 41598_2023_33993_MOESM3_ESM.pptx]

## Slide 1
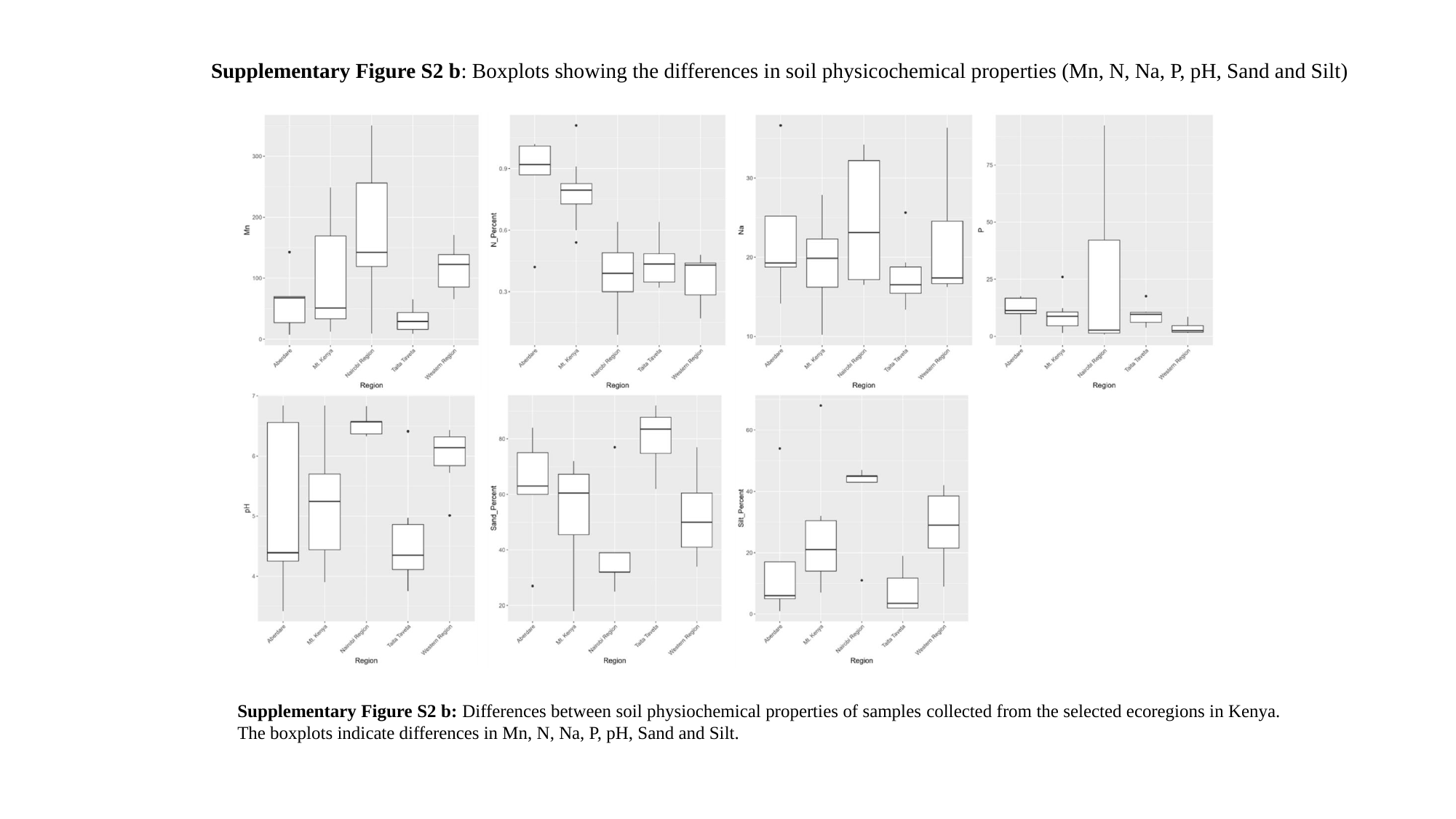

Supplementary Figure S2 b: Boxplots showing the differences in soil physicochemical properties (Mn, N, Na, P, pH, Sand and Silt)
Supplementary Figure S2 b: Differences between soil physiochemical properties of samples collected from the selected ecoregions in Kenya. The boxplots indicate differences in Mn, N, Na, P, pH, Sand and Silt.
